# Editorial: Cellular and synaptic mechanisms in the auditory system in health and disease

**DOI:** 10.3389/fncel.2025.1717740

**Published:** 2025-10-23

**Authors:** Bernhard Englitz, Christian Keine

**Affiliations:** 1 Computational Neuroscience Lab, Donders Center for Neuroscience, Radboud University, Nijmegen, Netherlands; 2 Division of Physiology, School of Medicine and Health Sciences, Carl von Ossietzky Universität Oldenburg, Oldenburg, Germany; 3 Research Center Neurosensory Science, Carl von Ossietzky Universität, Oldenburg, Germany

**Keywords:** auditory system, hearing, synaptic transmission, cellular mechanisms, synapse

## Introduction

The auditory system enables animals to navigate and interpret their acoustic environment through sophisticated neural computations. This process begins with the conversion of sound waves into electrical signals in the cochlea and culminates in complex encoding of location and context in central brain regions. Each stage relies on specialized cellular and synaptic mechanisms. Understanding these mechanisms, their development, changes with experience and age and susceptibility to disease, is key to comprehending both normal hearing and auditory dysfunction. This Research Topic explores the cellular and synaptic specializations underlying hearing, focusing on the resilience of synaptic transmission, developmental and age-related changes, and the modulatory influences that fine-tune auditory circuits. By bringing together diverse methods and perspectives across different processing stages, the studies in the present Research Topic contribute to a refined picture of the molecular and cellular machinery required for proper auditory function and its vulnerabilities, advancing the state of the art.

## Specialization in the auditory system

The peripheral auditory system employs sophisticated preprocessing to optimize neural encoding. In the cochlea, the vast dynamic range of audible sounds is compressed by three orders of magnitude for inner hair cell transduction (Rhode, 2007). Kondylidis et al., using optical coherence tomography, demonstrate that this compression occurs progressively along the basilar membrane in an intensity-dependent manner, optimizing the signal for neural transduction.

As signals ascends to the brainstem, they are further processed by synapses adapted for speed and temporal precision. As reviewed by Keine and Englitz, these synapses share common features, including large presynaptic terminals, fast-gating AMPA receptors, and a repertoire of voltage-gated ion channels that enable high-frequency synaptic transmission.

This high-frequency firing, however, places immense metabolic demands on the system, particularly for replenishing synaptic vesicles (SV). Addressing this challenge, Pizzi et al. explore the metabolic cost of this precision at an inhibitory brainstem synapse critical for sound localization. Their findings reveal that even the fundamental process of SV refilling requires auxiliary mechanisms beyond the canonical vesicular ATPase proton pump to keep pace with the relentless demand. This suggests that sustaining high-frequency signaling requires unique, and perhaps still undiscovered, molecular solutions.

## Ototoxicity, aging and hearing loss

Despite its sophisticated design, the auditory system is vulnerable to trauma and aging (Howarth and Shone, 2006; Kujawa and Liberman, 2009; Gold and Bajo, 2014). Cochlear synaptopathy, or ‘hidden hearing loss', involves damage to the synapse between inner hair cells (IHC) and spiral ganglion neurons occurring before hair cell loss. This explains why individuals with normal audiograms may struggle to hear in noisy environments.

Environmental toxins pose another significant threat, particularly for children and pregnant women (Tiwari et al., 2012; Olufemi et al., 2022). Bhatia et al. provide compelling evidence that chronic lead exposure induces cochlear synaptopathy and synapse loss without affecting outer hair cells (OHC). Through proteomic analysis, they identified proteins involved in the synaptic vesicle cycle as key targets of lead-induced ototoxicity.

Noise trauma, a common insult, can inflict similar damages and is particularly prominent in elderly individuals (Cunningham and Tucci, 2017). Oestreicher et al. show that even a single moderate noise exposure causes significant and persistent loss of IHC ribbon synapses in the high-frequency area of the cochlea. Intriguingly, they found that the remaining synapses compensated this loss by increasing their neurotransmitter release, indicating the presence of a potent compensatory mechanism and suggesting that ribbon counts alone may not fully predict synaptic output.

Further exploring the dose-dependent damage of acoustic trauma leading to cochlear synaptopathy, Blum et al. demonstrate that presynaptic ribbons are more susceptible to noise trauma than their postsynaptic partners, identifying the presynaptic element as the more fragile component of this first auditory synapse.

These studies naturally lead to the question of therapeutic intervention. Hemachandran et al. explored the potential to promote cochlear synapse regeneration after excitotoxic or noise-induced damage. They demonstrate both *in vitro* and *in vivo* that stimulating cyclic AMP signaling effectively promotes the regeneration of IHC ribbon synapses and restores partial function. This work identifies the cAMP/PKA pathway as a promising target for minimally invasive therapies aimed at reversing cochlear synaptopathy.

Complementing this outlook, the mini-review by Slika and Fuchs summarizes the powerful genetic tools, from viral vectors to CRISPR, now being used to study and potentially manipulate the olivocochlear efferent system for therapeutic gain.

## Development, maintenance, and plasticity

A central question in developmental neuroscience is the interplay between genetically encoded programs and activity-dependent refinement in shaping neural circuits. Challenging long-held assumptions about activity-dependence (Hubel and Wiesel, 1964; Blankenship et al., 2009; Kirkby et al., 2013; Wang and Bergles, 2015), Lessle et al. investigate the maintenance of the calyx of Held, a giant presynaptic terminal in the medial nucleus of the trapezoid body (MNTB) known for its temporal fidelity (von Gersdorff and Borst, 2002; Borst and Soria van Hoeve, 2012; Joris and Trussell, 2018). By selectively silencing neurotransmission after synapse maturation, they found that its fundamental structure, including active zone number and postsynaptic AMPAR composition, remained remarkably intact even after weeks of inactivity. This suggests the existence of a robust, genetically encoded program for maintaining this highly specialized synapse, largely independent of ongoing activity.

This inherent stability, however, is fine-tuned by other factors during development. While MNTB neurons typically receive a single calyx of Held input in adults, multiple calyces make contact to MNTB neurons during development (Hoffpauir et al., 2006; Rodríguez-Contreras et al., 2008; Holcomb et al., 2013; Sierksma et al., 2017). Chokr et al. explored the role of the classical complement cascade, a part of the innate immune system, in pruning and elimination of immature calyceal inputs. They found that C1q, the initiating molecule of this cascade, is expressed by microglia during the period of synapse elimination. While its absence did not prevent pruning, it resulted in a subtle but significant speed-up of auditory signal transmission, pointing to a nuanced role for immune-related molecules in fine-tuning auditory processing.

Developmental insults can have profound and lasting consequences on the auditory system. Mansour and Kulesza investigated the consequences of *in utero* exposure to valproic acid (VPA), an animal model relevant to autism spectrum disorder (ASD), which is often associated with auditory dysfunction (Moore et al., 2000; Bromley et al., 2013; Hernández-Díaz et al., 2024; Pack et al., 2024; Bolton et al., 2012; Christensen et al., 2013; Ramezani et al., 2019; Mansour et al., 2021). Their work reveals the near-complete obliteration of a specific glycinergic projection from the MNTB to the auditory thalamus, highlighting the vulnerability of specific auditory pathways to developmental disruptions and pointing to potential thalamic processing deficits in this ASD model.

The balance between structural stability and plasticity continues into adulthood and is profoundly impacted by aging (Ingham et al., 1998; Syka, 2002; Ouda et al., 2015). Rosskothen-Kuhl et al. address how the age at the onset of deafness influences central auditory organization. Using Fos mapping after cochlear implant stimulation, they demonstrate that rats deafened as young adults show rapid degradation of brainstem tonotopy, resembling neonatally deafened animals. In contrast, rats deafened as adults largely preserved their tonotopic organization. This suggests that the reduced plasticity of the aging brain, while limiting adaptation, may also confer some resilience that protects established neural circuits from maladaptive reorganization following injury.

While age-related changes in IHC and spiral ganglion neurons are well documented, relatively little is known about the impact of age on the efferent system (Adams and Schulte, 1997; Suryadevara et al., 2001; Bovee et al., 2024). Steenken et al. provide a detailed analysis of age-related changes in the olivocochlear efferent system of the gerbil cochlea. While confirming an overall decline in efferent terminals, they found that innervation density remained largely intact, implying a parallel degeneration of efferents and their targets. Another key finding was the increased prevalence of “orphaned” OHCs (lacking efferent input) in aged animals, suggesting that medial olicocochlear degeneration may precede OHC loss.

The degradation of the peripheral auditory system during aging or following cochlear damage is often compensated by downregulation of inhibition in the central auditory system (Caspary et al., 2008; Richardson et al., 2012; Caspary and Llano, 2018). Mellott et al. focus on the inferior colliculus (IC), a major integration hub, uncovering a significant age-related upregulation of dense core vesicles (DCVs), particularly during middle age. As DCVs contain neuromodulators and neurotrophins, this finding suggests an increase in modulatory capacity, possibly to compensate for the declining peripheral input or GABAergic inhibition. Notably, terminals containing DCVs appeared to be preferentially spared from age-related synapse loss.

## Molecular and neuromodulatory complexity

A deeper understanding of auditory circuit function requires dissecting its immense molecular and chemical diversity. Using the power of next-generation RNA sequencing (Macaulay and Voet, 2014; Svensson et al., 2018), Maraslioglu-Sperber et al. provide a high-resolution molecular and functional profile of neurons in the mouse lateral superior olive (LSO). They successfully correlate transcriptomic signatures with electrophysiological properties, delineating the two principal neuron types: ascending principal neurons (pLSOs) and descending lateral olivocochlear (LOC) neurons. The identification of hundreds of differentially expressed genes, including novel markers and specific ion channel subunits, offers a powerful resource for dissecting LSO circuitry and function.

Beyond the primary excitatory and inhibitory transmitters, neurons in the auditory system are influenced by neuromodulators that shape their function (Burger and Kopp-Scheinpflug, 2022). The review by Zhang and Burger focusses the rapidly growing knowledge on cholinergic modulation throughout the auditory pathway, with an emphasis on brainstem and midbrain nuclei. They detail the diverse array of nicotinic and muscarinic receptors and their multifaceted roles in shaping fundamental auditory computations, from gain control and noise protection to synaptic plasticity, underscoring the dynamic chemical landscape that governs our perception of sound.

## Conclusion and future directions

Collectively, this diverse set of 16 studies helps to advance our understanding of the auditory brainstem from a simple series of relays into a highly dynamic and sophisticated processing hub. The Research Topic highlights a theme: The very specializations that enable reliable signal transmission with sub-millisecond precision go alongside profound vulnerabilities. Yet, this fragility is counterbalanced by numerous protective, modulatory, and plastic mechanisms that are themselves shaped by development, insult, and age.

The path forward is illuminated by the innovative approaches showcased in this Research Topic. The high-resolution molecular atlases generated by techniques like single-cell sequencing are invaluable for designing targeted pharmacological or genetic interventions. Similarly, a deeper understanding of the brain's own compensatory strategies could lead to therapies that move beyond simple acoustic amplification, aiming instead to protect and restore synaptic fidelity. The surprising resilience of giant synapses to inactivity, the nuanced roles of neuro-immune interactions, and the paradoxical effects of aging on plasticity all open new avenues of investigation. Ultimately, this Research Topic might help to move beyond treating the downstream consequences of hearing loss and instead learn to preserve, repair, and even enhance the remarkable artwork of precision that is found in the auditory system.
